# Supplementary material for: H105A peptide eye drops promote photoreceptor survival in murine and human models of retinal degeneration
Source: Commun Med (Lond). 2025 Mar 21;5:81. doi: 10.1038/s43856-025-00789-8 (PMC11928584; doi:10.1038/s43856-025-00789-8)
Supplement: Supplementary file 1 — Supplemental Material [file 43856_2025_789_MOESM1_ESM.pdf]

SUPPLEMENTARY INFORMATION

Supplementary Figure 1. Alexa Fluor 488 labeled 17-mer, H105A and R99A peptides.

a) **Standard curves of Alexa Flour-488 labeled peptides in retinal extracts.** Fluorescence levels of each labeled peptide (17-mer, H105A and R99A) were measured in retinal extracts from C57BL/6J mouse eyes. Solutions of labeled peptides were prepared at concentrations ranging from 0.0 to 0.0625 μg/ml, with a total volume of 30 μl added per well. Each data point represents the average of two measurements per concentration.

b) **Effect of daily eyedrops of Alexa Fluor-488 labeled peptides on ERG $_a$wave and $_b$wave retinal function of *rd10* mice.** ERGs were performed in *rd10* mice treated with eye drops of AlexaFl-488-17-mer, AlexaFl-488-H105A and AlexaFl-488-R99A, following the depicted scheme. Plots show amplitude (*y*-axis) as function of light intensity (cd/s.m^2, *x*-axis). Each data point represents the average ± SD, with three mice (n=3) per condition, analyzed by unpaired t-test. ### $p = 0.00173$ and #### $p < 0.0001$ indicate statistical significance between AlexaFl488-H105A and vehicle; ** $p = 0.0073$ and **** $p < 0.0001$ indicate significance between AlexaFl488-H105A and AlexaFl488-17-mer. Eye drops of labeled peptides AlexaFl-488-17-mer and AlexaFl-488-H105A improved retinal function as measured by ERG, while labeled AlexaFl-488-R99A showed no improvement, consistent with results from their unlabeled counterparts. RE and LE refer to the right and left eyes, respectively.

Supplementary Figure 1.

(a) Standard curve of AlexaFl488-17-mer, H105A and R99A peptides in mouse retinal extracts

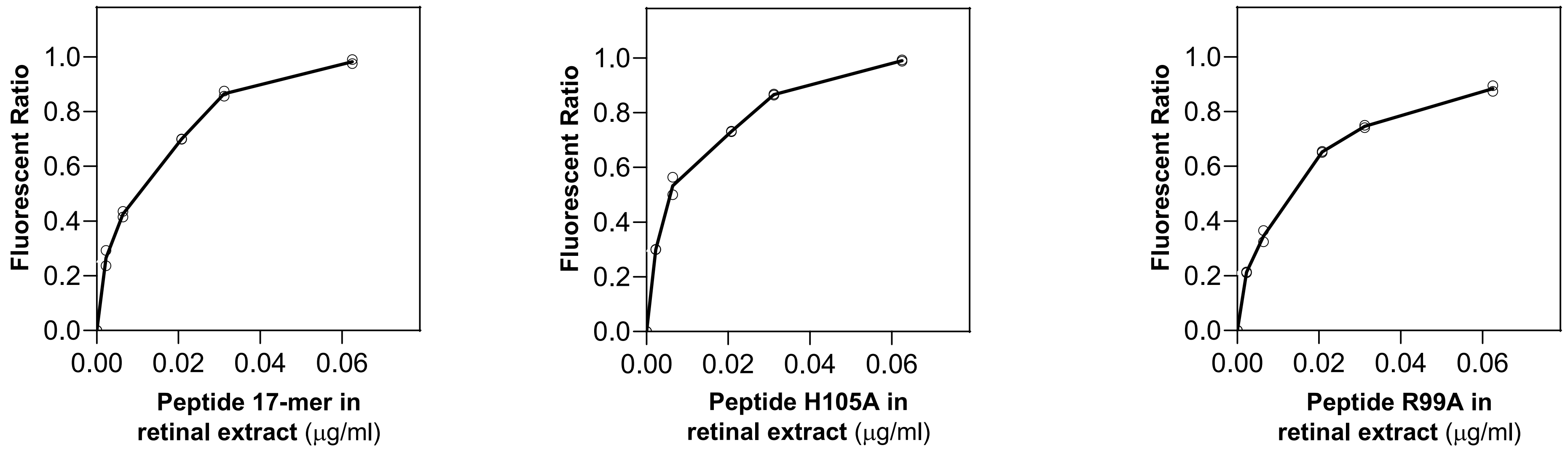

(b) Effect of daily eyedrops of labeled PEDF peptides on ERG a-wave and b-wave retinal function

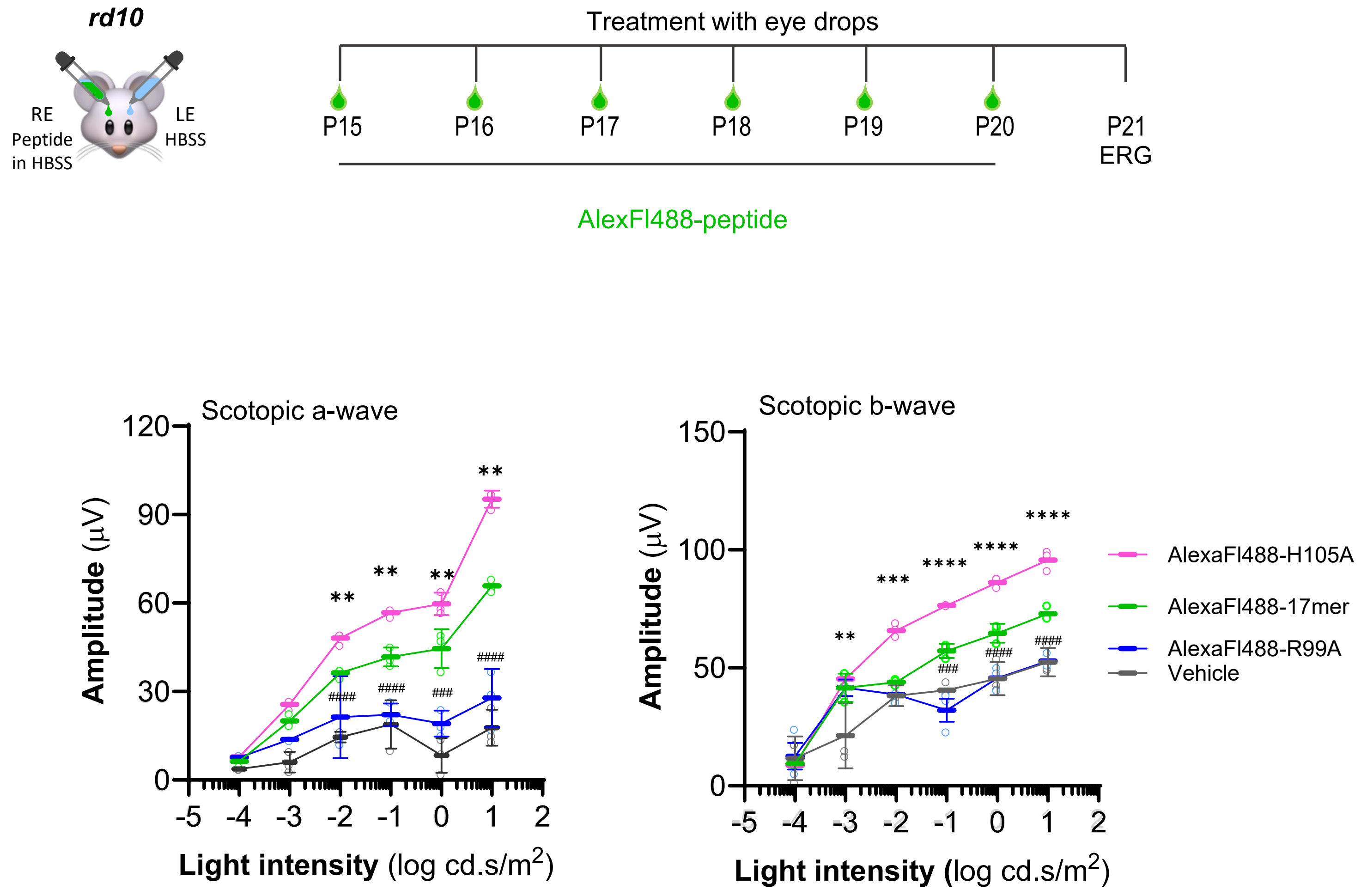

Supplementary Figure 2. ***In vivo*** **detection of dying photoreceptors in** ***rd10*** **mouse models by PS externalization monitoring.**

The highest PS externalization in fundi was at P17 for both models, being higher for *rd10* at all ages (**b**). The fluorescence slightly decreased at P21, to further decrease at P23 and being undetectable at P25. The fluorescence in mutant animals treated with HBSS or in wild type C57BL/6J mice was undetectable and considered as background controls. To correlate these observations with photoreceptor viability, we compared the height of the outer nuclear layer (ONL) of photoreceptor cells in the retina. The ONL of mice at P15 and P21 showed nuclei that likely corresponded to photoreceptor cells undergoing early apoptosis before their near or complete loss by P25 (**c**). Depletion of photoreceptors at P25 explained the decrease in PSVue® fluorescence in fundi (shown in **b**) and precluded use of PSVue® to detect dying cells at P25 for both models. The data agree with previous reports in which in the *rd10* and *rd10/Serpinf1*$^{-/-}$ mice, loss of photoreceptor phosphodiesterase activity leads to photoreceptor degeneration, starting at P16, and with near complete loss of photoreceptors by P25 and beyond[13].

Given that cell death is largely regulated by the BCL2 family of interacting proteins, such as BCL2-Associated X protein (BAX) that plays a central role in apoptosis and B-cell lymphoma 2 (BCL2) as an anti-apoptosis regulator, we evaluated their distribution in photoreceptors of our mutant mice at P21. Immunofluorescence of retinal cross sections was performed to detect BAX and BCL2. The photoreceptors of both models exhibited BAX, while BCL2 was undetectable (**d**). Thus, PSVue® fluorescence fundoscopy of mice agreed with detection of photoreceptors undergoing early apoptosis in *rd10* and *rd10/Serpinf1*$^{-/-}$ mice *in vivo*, highlighting the age of P21 as endpoint for the rest of the study.

a) Scheme showing the application of PSVue® 550 delivered via eye drops to *rd10* and *rd10/Serpinf1*$^{-/-}$ mice at P16, P20, P22 or P24. Eye drops of 5 µl of a solution of 1 mM PSVue® in HBSS were applied per eye and fluorescence fundoscopy was performed in live animals 24 hours after application. RE and LE refer to the right and left eyes, respectively.

b) *In vivo* detection of PS externalization in photoreceptors using PSVue®. Fluorescence fundoscopy micrographs of retinas at P17, P21, P23 and P25 of *rd10, rd10/Serpinf*1$^{-/-}$, and C57BL/6J mice after 24 h of administering PSVue® are shown. The right image on each row corresponds to retinas with HBSS (vehicle) only at P21, corresponding to background. Quantification of fluorescence intensity was done using ImageJ on images acquired by subtracting the background. Plots were generated using GraphPad. Each data point corresponds to the average of 3 images per retina for a total of five retinas (n = 5) per age.

c) Retinal cross sections stained with DAPI showing the distribution of nuclei in *rd10* and *rd10/Serpinf*1$^{-/-}$ mice between P15 and P25. Representative images are shown. The dotted lines demarcate the outer nuclear layer (ONL) for photoreceptor nuclei.

d) Retinal cross sections showing the distribution of BAX and BCL2 in *rd10* and *rd10/Serpinf*1$^{-/-}$ mice at P21 by immunofluorescence. Representative images are shown. For all immunofluorescence images shown, five retinas (n = 5) were used per group. ONL, outer nuclear layer, OS, outer segments, and IS, inner segments.

Supplementary Figure 2.

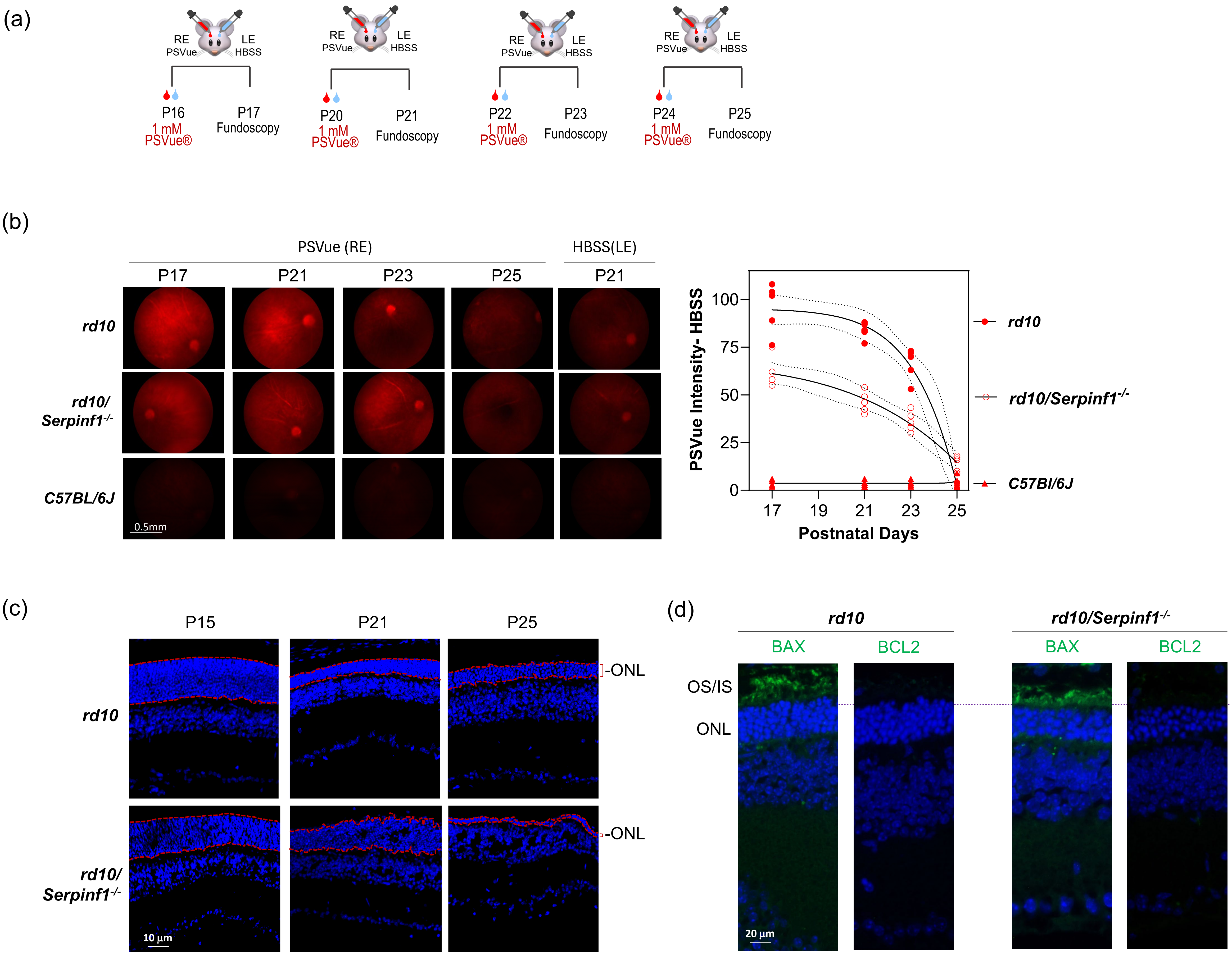

Supplementary Figure 3. PSVue labeling of phosphatidylserine (PS) externalization associated with photoreceptor cell death in the *rd10* retina.

Eye drops of PSVue® 480 (Molecular Targeting Technologies Inc, Catalogue number P-1003) were administered to *rd10* mice at P21 to detect PS externalization linked to photoreceptor cell death. A volume of 5 µl of 1 mM PSVue® 480 solution at 1 mM in HBSS was applied per eye, and 24 hours post-application, whole eyes were collected to examine retinal fluorescence. RE and LE refer to the right and left eyes, respectively.

For retinal cross sections, both eyes were aligned before enucleation to facilitate dorsal/ventral axis sectioning. Eyes were incised at the cornea, fixed in 2.5% glutaraldehyde for 20 min, followed by fixation in 10% neutral buffered formalin at 4°C for at least 48 h. Samples were then paraffin embedded, sectioned and imaged by fluorescence microscopy. Images were acquired using a ZEISS 700 confocal microscope at 20X.

Fluorescent micrographs show retinal cross-sections from *rd10* mice treated with PSVue 480 eye drops (1mM) at postnatal day 21 (P21). PSVue 480 labeling (green) highlights phosphatidylserine (PS) externalization localized to the outer nuclear layer (ONL) and outer segments (OS), aligning with previous studies in RCS rats[36]. Nuclei were counterstained with DAPI (blue). Four retinas (n = 4) per group were analyzed. Scale bar = 20 µm.

Supplementary Figure 3.

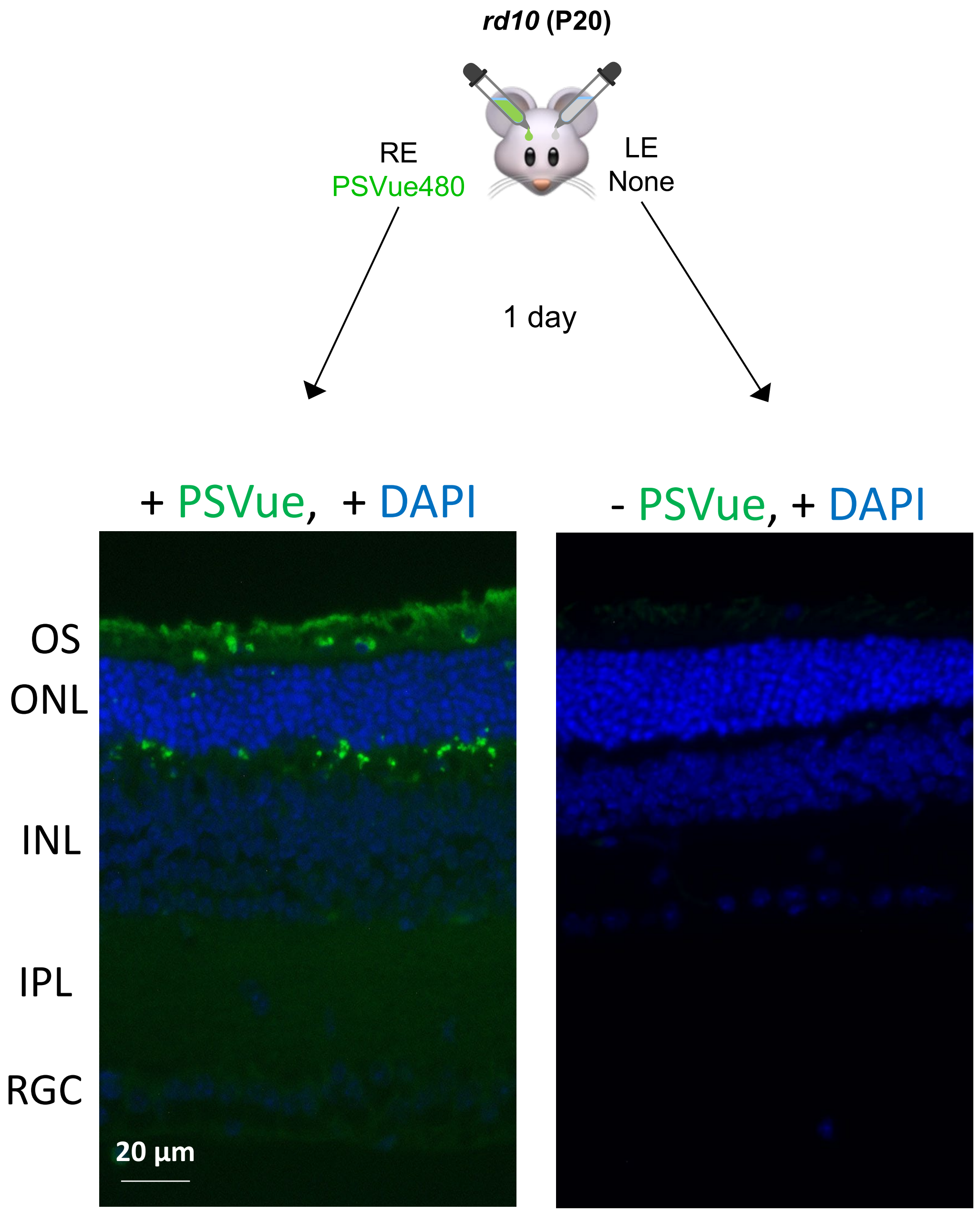

Supplementary Figure 4. Sustained Expression of H105A Delivered by AAV2 in Transduced Retinas Over Six Months

As outlined in the scheme of **Figure 7a**, each *Rho*$^{P23H/+}$ mouse received an intravitreal injection (IVT) of AAV-H105A in one eye and AAV-GFP, a control virus expressing GFP, in the contralateral eye at P5. At P19 and P180, the mRNA and protein were assessed.

(**a**) RT-PCR amplicons of H105A mRNA (120 bp) from transduced retinas, confirming viral mRNA expression at P19 and P180. Amplicons migrating at 270 bp correspond to AAV-H105A genomic DNA.

(**b)** GFP expression in transduced retinas: a fundoscopy image of an AAV-GFP-transduced retina at P19 reveals uniform transduction across the retinal tissue. Cross-sections of murine retinas at P19 and P180 display GFP fluorescence (green) across all retinal layers, with DAPI-staining of nuclei (blue) and rhodopsin (red, labeling of photoreceptors) confirmed that the AAV-GFP IVT primarily transduced ganglion cells and Müller glia cells.

(**c**) We also utilized a custom anti-H105A antibody labeled with a FLAG-tag and validated its specificity in COS7 cells transduced with AAV-H105A. H105A production in transduced COS7 cells was confirmed by the specific staining (red, white arrows), with signals appearing in the secretory pathway. No signal was observed in control images without primary antibodies (with anti-FLAG and Alexa Fluor 568 anti-mouse secondary and tertiary antibodies, respectively).

(**d**) H105A expression following AAV2-H105A IVT was revealed by specific staining with anti-H105A (red) in P180 *Rho*$^{P23H/+}$ mouse retinas compared to control AAV-GFP eyes. H105A was detected across the retina, including both dorsal and ventral regions, with prominent signals in the ganglion cell layer (GC) and in photoreceptor inner segment (IS). INL: inner nuclear layer, ONL: outer nuclear layer.

Supplementary Figure 4.

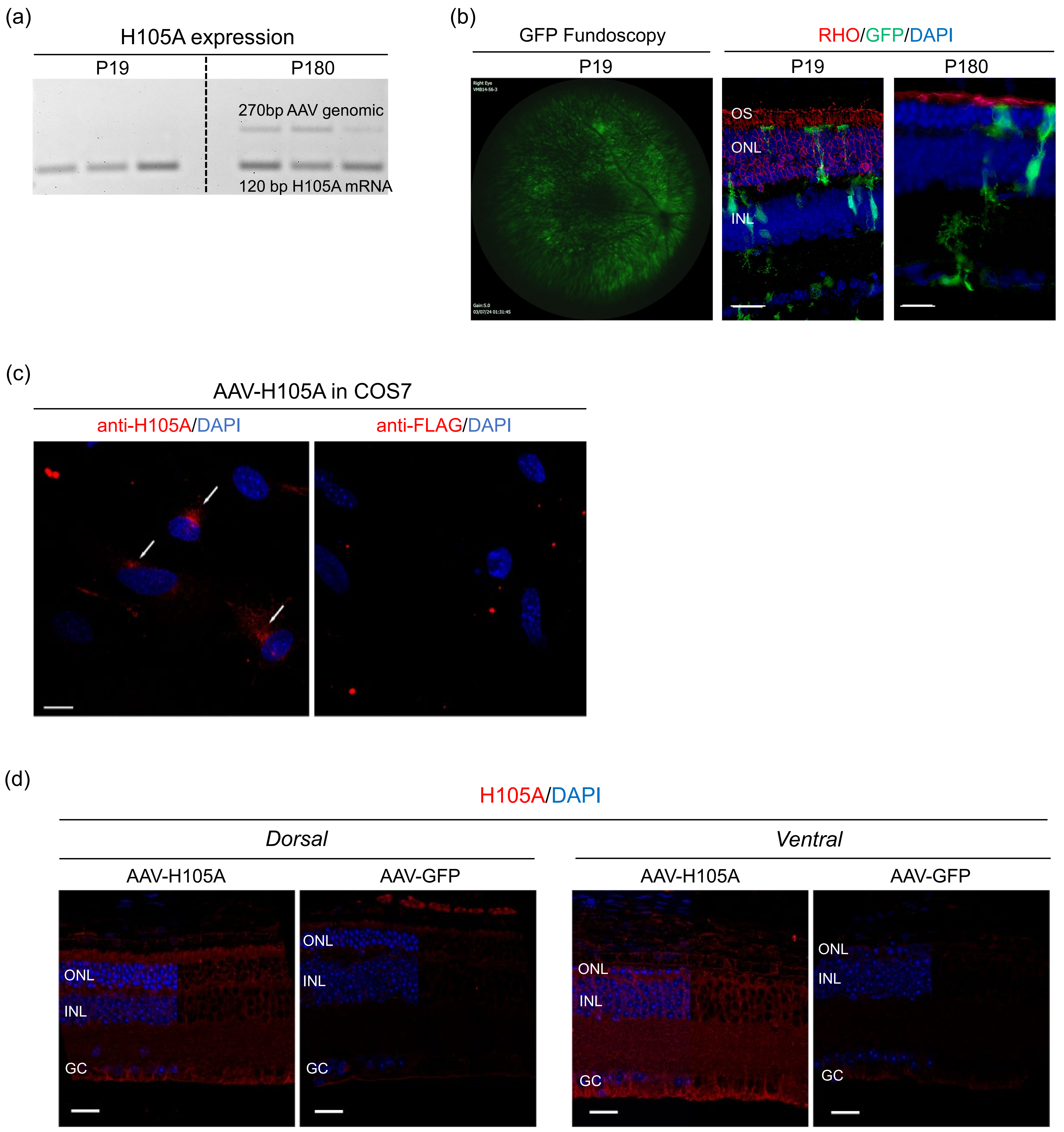

Supplementary Figure 5. H105A localization in AAV transduced retinas and eye drops treated retinas

The distribution of H105A (green) in $Rho^{P23H/+}$ retinas was visualized using a custom anti-H105A antibody as described in Methods. Following intravitreal injection (IVT) of AAV-H105A at P5, H105A was detected throughout all retinal layers, specifically the photoreceptor inner segments and outer segments (IS/OS) at both P19 (**a**) and P180 (**e**). Control retinas treated with AAV-GFP showed no signal at either P19 (**b**) or P180 (**f**).

Daily eye-drop-delivered H105A between P14 and P19 penetrated the retina, reached the photoreceptors, and was detected throughout the retina, mostly in the IS/OS (**c**). No specific staining was observed at P19 in retinas treated with vehicle (HBSS) eye drops (**d**).

(g-i) Negative controls for immunostaining. No staining was detected in untreated $Rho^{P23H/+}$ retinas at P19 (**g**), in sections where the antibody was pre-incubated with 400 µM H105A peptide for one hour (**Panel h**), or in sections without the primary antibody (**i**). Nuclei were stained with DAPI and pseudocolored in white. Scale bar: 20 µm. INL, inner nuclear layer; IS/OS, inner/outer segments; ONL, outer nuclear layer.

Supplementary Figure 5.

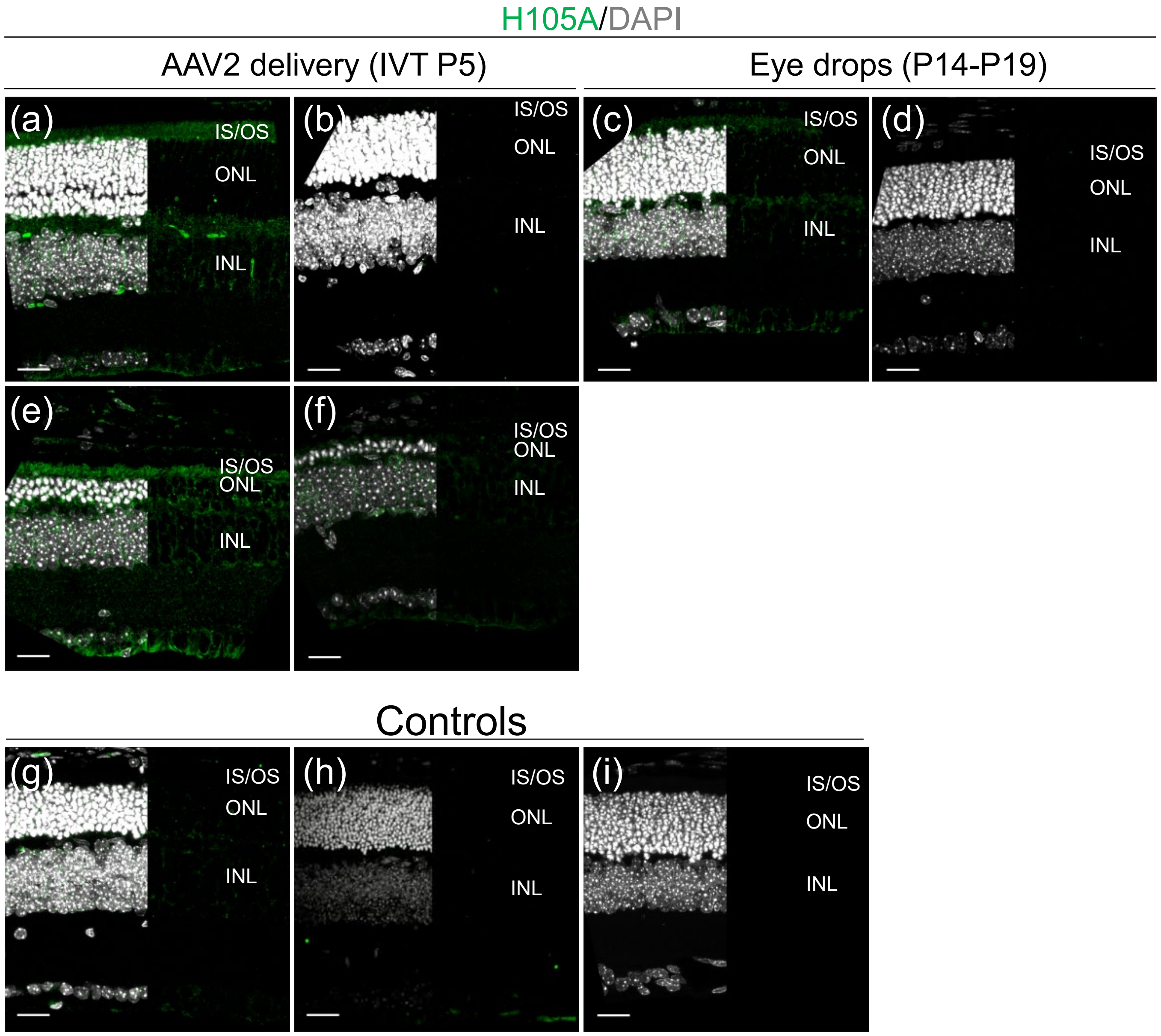

Supplementary Figure 6. Live retinal organoid staining with cell death indicator dyes.

Retinal organoids were treated for 24 hours with 500 µg/ml CSE or with vehicle (DMSO) for control. Photomicrographs illustrate bright field and fluorescence images of whole-mount, live ROs incubated with ethidium homodimer, which labels the nuclei of dead cells (**a**), or PsVue794, which labels PS externalization, an early event in apoptosis (**b**). Scale bars represent 100 µm and apply to all images.

Supplementary Figure 6.

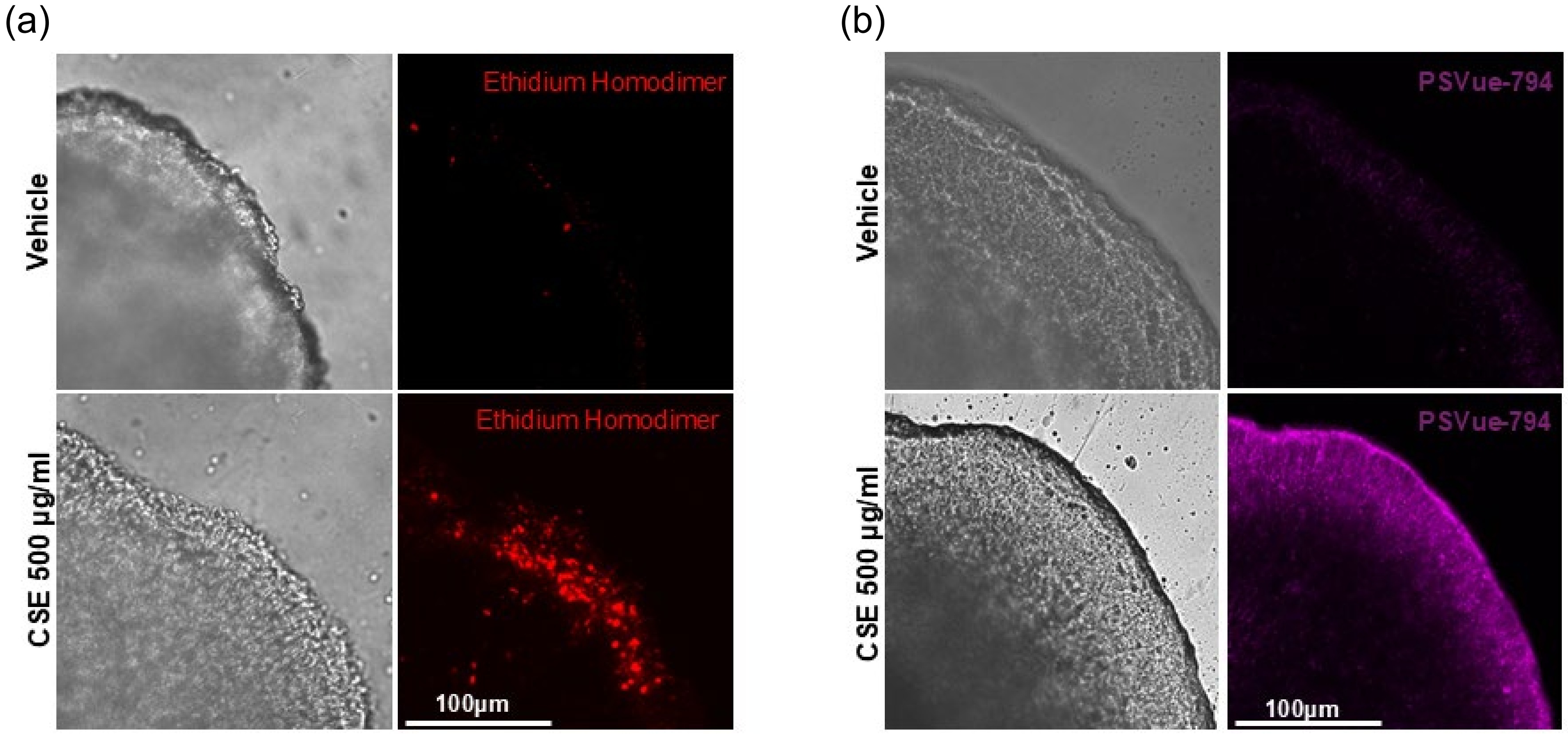

Supplementary Figure 7. Effects of extending the timeline or administering eye drops of 17-mer[H105A] peptide every other day in *rd10* mice.

Eye drops of H105A peptide solution were administered to *rd10* mice either daily (**a**) or every other day (**d**). A total of 5 µl of a solution containing H105A peptide at 1 mg/ml in HBSS were applied as eye drops. The vehicle HBSS was added as eye drops in the contralateral eyes. When eye drops were added daily starting at P15 until P24, we noticed a rescue in the ONL thickness in *rd10* mice (**b**)**.** Eye drops also improved rod photoreceptor function in ERG in response to low intensity light at P25 (**c**). When eye drops of H105A were added every other day, a rescue in the ONL thickness in *rd10* mice compared to vehicle was also observed (**e**), with an improvement in a-wave of ERG, but no significant effect on the b-wave at P25 (**f**). The data demonstrate the efficacy of peptide H105A eye drops either daily or every other day in an extended timeline to stabilize photoreceptor morphology and light response function loss in *rd10* mice, however, these were less efficacious than daily H105A peptide eye drops.

a) Scheme showing a timeline of peptide administration via daily eye drops of H105A peptide to *rd10* mice between P15 and P24. Assays were performed at P25. RE and LE refer to the right and left eyes, respectively.

b) Representative microphotographs of retinal cross sections of *rd10* treated as in **Panel a** and stained with hematoxylin and eosin are shown. To the right, a spider plot illustrating the thickness of the ONL of *rd10* mice treated with or without H105A as a function of distance from the optic nerve (ON). For all histology shown, five retinas (n=5) per group were evaluated and each data point corresponds to the average ± SD per location relative to the ON by paired t-test. Statistical significance between H105A and untreated is indicated by ***p = 0.00173, ****p < 0.0001.

c) ERG of *rd10* mice at P25 and treated as in **Panel a** were performed. Plots show amplitude (*y*-axis) for a- and b-wave as function of light intensity (cd/s.m^2, *x*-axis). For ERG data shown, the number of mice evaluated were five for *rd10* (n=5) and each data point corresponds to the average ± SD by paired t-test. Statistical significance between H105A and untreated is indicated by ***p = 0.00173, ****p < 0.0001.

d) Scheme showing a timeline of peptide administration via eye drops of H105A peptide to *rd10* mice starting at P15 every other day until P23. Assays were performed at P25.

e) Representative microphotographs of retinal cross sections of *rd10* treated as in **Panel d** and stained with hematoxylin and eosin are shown. To the right, a spider plot illustrating the thickness of the ONL of *rd10* mice treated with or without H105A as a function of distance from the optic nerve (ON). For all histology shown, five retinas (n=5) per group were evaluated and each data point corresponds to the average ± SD per location relative to the ON by paired t-test. Statistical significance between H105A and untreated is indicated by **p ≤ 0.009, ***p = 0.00173, ****p < 0.0001.

f) ERG of *rd10* mice at P25 and treated as in **Panel d**. Plots show amplitude (*y*-axis) for a- and b-wave as function of light intensity (cd/s.m^2, *x*-axis). For ERG data shown, the number of mice evaluated were five (5) for *rd10* and each data point corresponds to the average ± SD by paired t-test. Statistical significance between H105A and untreated is indicated by ***p = 0.00173, ****p < 0.0001.

Supplementary Figure 7.

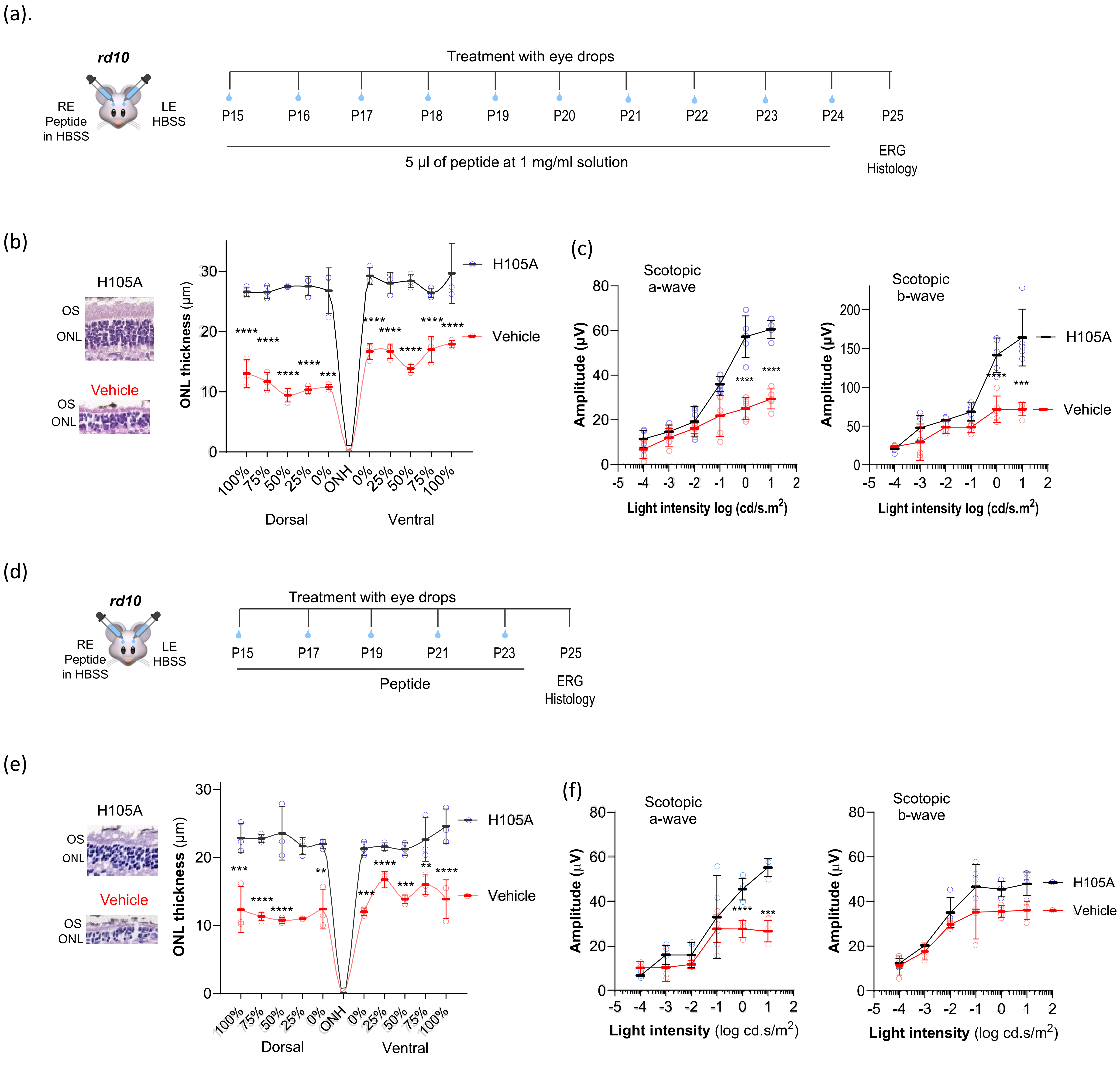

Supplementary Figure 8. Lack of efficacy of 29-mer and 29-mer[H105A] peptides in delaying photoreceptor cell death

The PEDF-derived peptide 29-mer (positions 93-121 of human PEDF), previously shown to prevent dry eye in corneal models when administered via eye drops[62], was modified to create two peptides: 29-mer and 29-mer[H105A]. These peptides contain either the 17-mer or H105A sequences in the central region (highlighted in yellow in Panel a). To assess their ability to penetrate the retina and protect against photoreceptor cell death, we administered daily eye drops of 5 µl of 29-mer or 29-mer[H105A] at 1 mg/ml in HBSS to the left eyes of *rd10* and *rd10/Serpinf1*$^{-/-}$ mice, with the contralateral eyes receiving HBSS as a vehicle control, starting at P15 (**a**).

At P21, PSVue 550 fluorescence fundoscopy revealed no reduction in photoreceptor cell death in either in *rd10* and *rd10/Serpinf1*$^{-/-}$ mice with 29-mer of 29-mer[H105A] compared to the vehicle (**b**). To further evaluate retinal penetration, we administered chemically synthesized 29-mer and 29-mer[H105A] peptides conjugated with Alexa Fluor™ 488 dye (Alexa 488) to the left eyes of C57BL/6J mice, with HBSS applied to the right eye as control. Fluorescence fundoscopy conducted one hour post-delivery showed no fluorescence in the posterior retina for either peptide, similar to the control eyes (**c**).

These results indicate that both 29-mer and 29-mer[H105A], when delivered by eye drops, failed to penetrate the retina and were ineffective in protecting the photoreceptors of *rd10* mice.

a) Scheme showing a timeline of daily peptide administration via eye drops to *rd10* and *rd10*/*Serpinf*1 null mice between P15 and P21. The sequences of the peptides are given, and the yellow highlight corresponds to the 17-mer and 17-mer[H105A] region in the 29-mer and 29-mer[H105A], respectively.

b) Mice were treated with peptides 29-mer and 29-mer[H105A] as indicated in **Panel a**. Representative fluorescence fundoscopy micrographs of *rd10* and *rd10/Serpinf*1$^{-/-}$ retinas exposed to PSVue® are shown. Quantification of fluorescence intensity was performed using ImageJ of images acquired by subtracting the background of images of mice treated HBSS without PSVue®. Plots were generated using GraphPad. Each data point corresponds to the average of 3 ROIs per retina for a total of three retinas (n = 3) per genotype. Scale bar indicates 0.5 mm.

c) AlexaFluor488-labeled 29-mer and AlexaFluor488-29-mer[H105A] were administered to C57BL/6J mice at P21 days of age via eye drops (5 µl of a solution of each peptide at 1 mg/ml in HBSS per eye). Micrographs of fluorescence fundoscopy performed *in vivo* of the posterior retinas are shown. Scale bar indicates 0.5 mm.

Supplementary Figure 8.

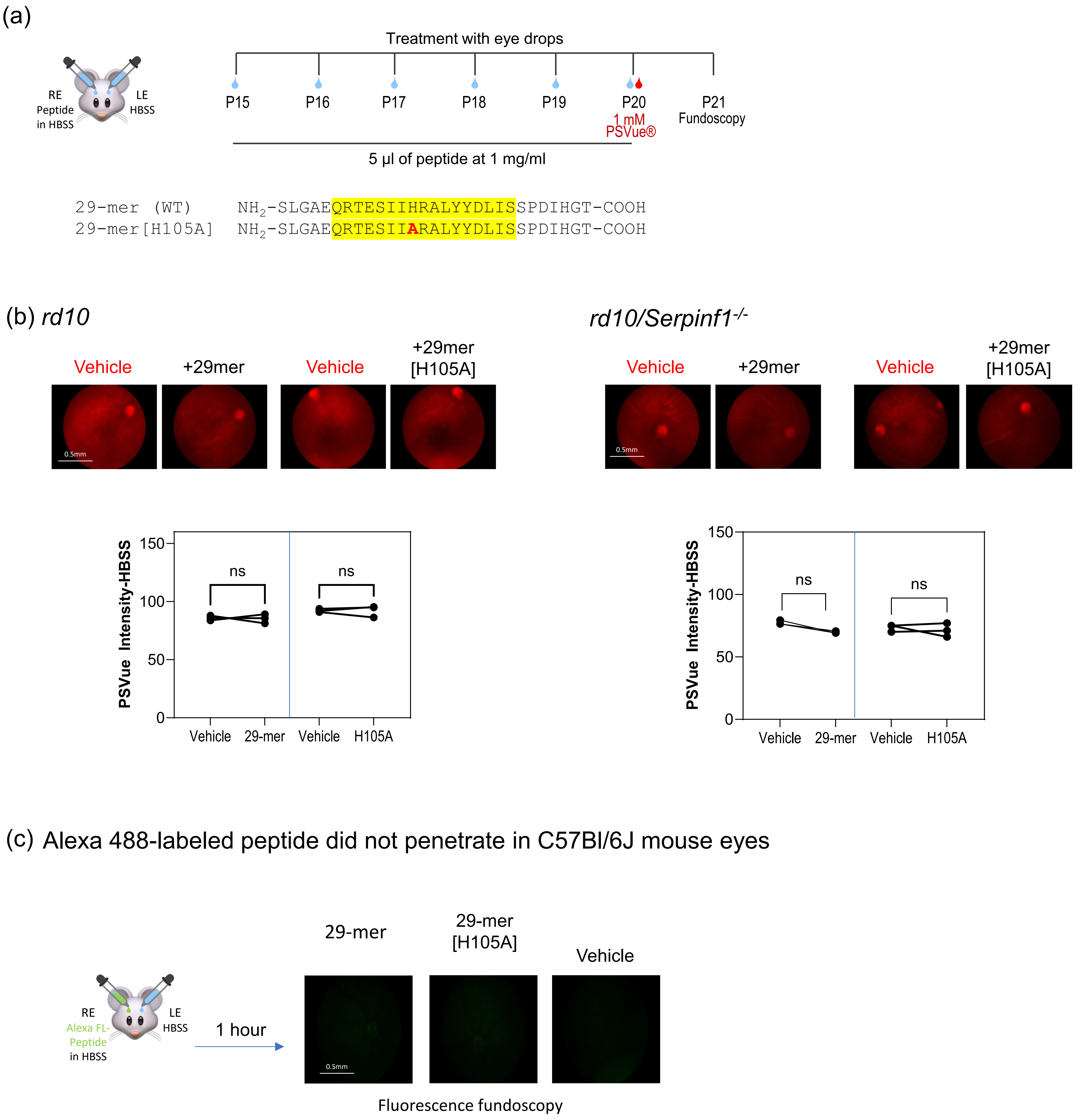

Supplementary Table 1. Peptides used in the study.

Peptide	Sequence
17-mer	NH_2-QRTESIIHRALYYDLIS-COOH
H105A	NH_2-QRTESII**A**RALYYDLIS-COOH
R99A	NH_2-Q**A**TESIIHRALYYDLIS-COOH
29-mer	NH_2-SLGAEQRTESIIHRALYYDLISSPDIHGT-COOH
29-mer[H105A]	NH_2-SLGAEQRTESII**A**RALYYDLISSPDIHGT-COOH
AlexaFl-488-17-mer	AlexaFl-QRTESIIHRALYYDLIS-COOH
AlexaFl-488-H105A	AlexaFl-QRTESII**A**RALYYDLIS-COOH
AlexaFl-488-R99A	AlexaFl-Q**A**TESIIHRALYYDLIS-COOH
AlexaFl-488-29-mer	AlexaFl-SLGAEQRTESIIHRALYYDLISSPDIHGT-COOH
AlexaFl-488-29-mer[H105A]	AlexaFl-SLGAEQRTESII**A**RALYYDLISSPDIHGT-COOH

*A in bold indicates an alteration to alanine from the 17-mer sequence

Supplementary Table 2. Stability of 17-mer[H105A] peptide.

	Concentration (mg/mL) Day1	Store	Concentration (mg/mL) Day8
1	1.04	RT	1.08
2	1.04	4 ˚C	1.16
3	1.04	-20 ˚C	1.08
4	1.04	-20 ˚C [FT]*	1.28

*FT, Freeze and thaw cycles daily

Supplementary Table 3. Antibodies used in the study.

**Antibody**	**Type & host**	**Application**	**Dilution**	**Company, catalog number**
Anti-Bcl-2	Rabbit Polyclonal	IF	1:100	Abcam, ab196495
Anti-Bax	Rabbit Monoclonal	IF	1:200	Cell signaling, 14796
Alexa Fluor 488	Goat anti-Rabbit IgG (H+L)	IF	1:200	Thermo Fisher Scientific, A-32731
Anti-RHO 1D4	Mouse Monoclonal	IF	1:500	Santa Cruz, sc-57432
Anti-GFP	Rabbit Polyclonal	IF	1:200	Thermo Fisher Scientific, A11122
Anti-H105A	HuCAL, Monoclonal	IF	1:90	BioRad, custom
Anti-FLAG M2	Mouse Monoclonal	IF	1:100	Merck, F1804
Alexa Fluor 568	Goat anti-Mouse IgG (H+L)	IF	1:1000	Thermo Fisher Scientific, A-11004
Anti-Opsin Blue	Rabbit Polyclonal	IF	1:200	Merck, AB5407
Anti-Opsin Red/Green	Rabbit Polyclonal	IF	1:200	Merck, AB5405
Anti-Iba1	Rabbit Polyclonal	IF	1:100	Wako; 019-19741

*IF, immunofluorescence
