# Supplementary material for: H105A peptide eye drops promote photoreceptor survival in murine and human models of retinal degeneration
Source: Commun Med (Lond). 2025 Mar 21;5:81. doi: 10.1038/s43856-025-00789-8 (PMC11928584; doi:10.1038/s43856-025-00789-8)
Supplement: Supplementary file 2 — Description of Additional Supplementary Files [file 43856_2025_789_MOESM2_ESM.pdf]

Description of additional supplementary files

File name: Supplementary Data 1
Description: An EXCEL file containing source numerical data
