# Supplementary material for: H105A peptide eye drops promote photoreceptor survival in murine and human models of retinal degeneration
Source: Commun Med (Lond). 2025 Mar 21;5:81. doi: 10.1038/s43856-025-00789-8 (PMC11928584; doi:10.1038/s43856-025-00789-8)
Supplement: Supplementary file 4 — REPORTING SUMMARY [file 43856_2025_789_MOESM4_ESM.pdf]

nature portfolio

Corresponding author(s): DAPR, manuscript COMMSMED-24-0643B

Last updated by author(s): Feb 1, 2025

Reporting Summary

Nature Portfolio wishes to improve the reproducibility of the work that we publish. This form provides structure for consistency and transparency in reporting. For further information on Nature Portfolio policies, see our Editorial Policies and the Editorial Policy Checklist.

Statistics

For all statistical analyses, confirm that the following items are present in the figure legend, table legend, main text, or Methods section.

n/a	Confirmed	
☐	☒	The exact sample size (*n*) for each experimental group/condition, given as a discrete number and unit of measurement
☐	☒	A statement on whether measurements were taken from distinct samples or whether the same sample was measured repeatedly
☐	☒	The statistical test(s) used AND whether they are one- or two-sided<br>*Only common tests should be described solely by name; describe more complex techniques in the Methods section.*
☐	☒	A description of all covariates tested
☐	☒	A description of any assumptions or corrections, such as tests of normality and adjustment for multiple comparisons
☐	☒	A full description of the statistical parameters including central tendency (e.g. means) or other basic estimates (e.g. regression coefficient) AND variation (e.g. standard deviation) or associated estimates of uncertainty (e.g. confidence intervals)
☐	☒	For null hypothesis testing, the test statistic (e.g. *F*, *t*, *r*) with confidence intervals, effect sizes, degrees of freedom and *P* value noted<br>*Give P values as exact values whenever suitable.*
☒	☐	For Bayesian analysis, information on the choice of priors and Markov chain Monte Carlo settings
☒	☐	For hierarchical and complex designs, identification of the appropriate level for tests and full reporting of outcomes
☒	☐	Estimates of effect sizes (e.g. Cohen's *d*, Pearson's *r*), indicating how they were calculated

Our web collection on statistics for biologists contains articles on many of the points above.

Software and code

Policy information about availability of computer code

Data collection	Microsoft EXCEL, Image J, and NIKON software for images
Data analysis	GraphPad 10.2.2

For manuscripts utilizing custom algorithms or software that are central to the research but not yet described in published literature, software must be made available to editors and reviewers. We strongly encourage code deposition in a community repository (e.g. GitHub). See the Nature Portfolio guidelines for submitting code & software for further information.

Data

Policy information about availability of data

All manuscripts must include a data availability statement. This statement should provide the following information, where applicable:

- Accession codes, unique identifiers, or web links for publicly available datasets
- A description of any restrictions on data availability
- For clinical datasets or third party data, please ensure that the statement adheres to our policy

All data are available in the main text or the supplementary materials. All data are represented in all the figures and a Meta data file has been submitted with all the individual raw data for this manuscript.

Research involving human participants, their data, or biological material

Policy information about studies with human participants or human data. See also policy information about sex, gender (identity/presentation), and sexual orientation and race, ethnicity and racism.

Reporting on sex and gender	N/A
Reporting on race, ethnicity, or other socially relevant groupings	N/A
Population characteristics	N/A
Recruitment	N/A
Ethics oversight	N/A

Note that full information on the approval of the study protocol must also be provided in the manuscript.

Field-specific reporting

Please select the one below that is the best fit for your research. If you are not sure, read the appropriate sections before making your selection.

☒ Life sciences ☐ Behavioural & social sciences ☐ Ecological, evolutionary & environmental sciences

For a reference copy of the document with all sections, see nature.com/documents/nr-reporting-summary-flat.pdf

Life sciences study design

All studies must disclose on these points even when the disclosure is negative.

Sample size	We did not select either males or females in our studies because the murine models here used have not been reported to show differences in the progression of the disease between sexes, as also reported in patients with mutations in the PDE6 enzyme or P23H mutation in rhodopsin. Therefore, based on the “reduction" 3R requirement for animal studies, we did not exclude animals from the study, and all the bred animals participated in the study.
Data exclusions	No data exclusion.
Replication	Each experiment was replicated at least three biological times.
Randomization	We did not choose a particular animal, and this should be consider randomization, as we did it in different cages and times. Each experiment is done based on age and genotype.
Blinding	One scientist performed all the experiments, and the genotype of the animals is known. It will be hard to not be aware of which animal receives treatment or is control as we kept left eye for treatment and right eye for control, to make it easier for the scientist.

Reporting for specific materials, systems and methods

We require information from authors about some types of materials, experimental systems and methods used in many studies. Here, indicate whether each material, system or method listed is relevant to your study. If you are not sure if a list item applies to your research, read the appropriate section before selecting a response.

Materials & experimental systems

n/a	Involved in the study	
☐	☒	Antibodies
☐	☒	Eukaryotic cell lines
☒	☐	Palaeontology and archaeology
☐	☒	Animals and other organisms
☒	☐	Clinical data
☒	☐	Dual use research of concern
☒	☐	Plants

Methods

n/a	Involved in the study	
☒	☐	ChIP-seq
☒	☐	Flow cytometry
☒	☐	MRI-based neuroimaging

Antibodies

Antibodies used	Anti-Bcl-2 Rabbit Polyclonal (Abcam, ab196495)<br>Anti-Bax Rabbit Monoclonal (Cell Signaling, 14796)<br>Alexa Fluor 488 Goat anti-Rabbit IgG (H+L) (Fisher Scientific, A-32731)<br>Anti-RHO 1D4 Mouse Monoclonal (Santa Cruz, sc-57432)<br>Anti-GFP Rabbit Polyclonal (Thermo Fisher Scientific, A11122)<br>Anti-H105A HuCAL, Monoclonal (BioRad, custom)<br>Anti-FLAG M2 Mouse Monoclonal (Merck, F1804)<br>Alexa Fluor 568 Goat anti-Mouse IgG (H+L) I(Thermo Fisher Scientific, A-11004)<br>Anti-Opsin Blue Rabbit Polyclonal (Merck, AB5407)<br>Anti-Opsin Red/Green Rabbit Polyclonal (Merck, AB5405)<br>Anti-Iba1 Rabbit Polyclonal (Wako; 019-19741)<br>Anti-FLAG M2 (Merck, F1804)
Validation	nti-Bcl-2 Rabbit Polyclonal IF 1:100 Abcam, ab196495 . Antibody was validated by abcam chrome-extension://efaidnbmnnnibpcajpcglclefindmkaj/https://doc.abcam.com/datasheets/active/ab196495/en-us/bcl-2-antibody-ab196495.pdf in rats<br><br>Anti-Bax Rabbit Monoclonal IF 1:200 Cell signaling, 14796 The antibody was validate by cell signaling in rabbits https://www.cellsignal.com/products/primary-antibodies/bax-d3r2m-rabbit-mab/14796?srsltid=AfmBOop0YrFJe-vU4gBLxoWgb3povC0p0EyWA71NLNQ7tGPeDRyfoP7n<br><br>Anti-H105A custom HuCAL antibody was validated in this study by IF in H105A transfected and control transfected cells, by IF after competition with the antigen H105A peptide, IF in tissue transduced with AAV2 expressing H105A or AAV2 expressing GFP as control.

Eukaryotic cell lines

Policy information about cell lines and Sex and Gender in Research

Cell line source(s)	ATCC COS-7 cell line
Authentication	Cells were not autenticated
Mycoplasma contamination	Cells were tested for mycoplasm contamination and resulted negative
Commonly misidentified lines (See ICLAC register)	none

Animals and other research organisms

Policy information about studies involving animals; ARRIVE guidelines recommended for reporting animal research, and Sex and Gender in Research

Laboratory animals	Species: Mus musculus; lines rd10, rd10/Serpinf1-/- and RhoP23H/+.
Wild animals	The study did not involve wild animals.
Reporting on sex	We did not select either males or females in our studies because the murine models used have not been reported to show differences in the progression of the disease between sexes. We found no difference in response to the treatment in males or females. Sex based analysis was send to the reviewers as per their request.
Field-collected samples	No field collected.
Ethics oversight	All the experimental procedures were approved by the National Eye Institute Animal Care and Use Committee and the Ethical Committee of University of Modena and Reggio Emilia and by the Italian Ministero della Salute (150/2021-PR) and were performed as per guidelines of the Association for Research in Vision and Ophthalmology statement for the Use of Animals in Ophthalmic and Vision Research and in accordance with the ARRIVE guidelines.

Note that full information on the approval of the study protocol must also be provided in the manuscript.

Plants

Seed stocks	N/A
Novel plant genotypes	N/A
Authentication	N/A
